# Correction: Detecting face presentation attacks in mobile devices with a patch-based CNN and a sensor-aware loss function

**DOI:** 10.1371/journal.pone.0264409

**Published:** 2022-02-17

**Authors:** The PLOS ONE Staff

There are errors in the Funding statement. The publisher apologizes for the error. The correct Funding statement is as follows: This study was supported by Motorola Inc., BioLive, and FAPESP DejaVu (Grant #2017/12646-3) awarded to AR. The funders had no role in study design, data collection and analysis, decision to publish, or preparation of the manuscript.

A Competing Interest statement is missing. The correct Competing Interest statement is as follows: The authors have read the journal’s policy and have the following competing interests: AR received a grant from Motorola Inc. There are no patents, products in development or marketed products associated with this research to declare. This does not alter our adherence to PLOS ONE policies on sharing data and materials.

In the Author Contributions, “Investigation” should also be listed under Waldir R. Almeida’s contributions.
