# Supplementary material for: Detecting face presentation attacks in mobile devices with a patch-based CNN and a sensor-aware loss function
Source: PLoS One. 2020 Sep 4;15(9):e0238058. doi: 10.1371/journal.pone.0238058 (PMC7473524; doi:10.1371/journal.pone.0238058)
Supplement: Attachment — Submitted filename: 2020_biolive_pad_plosone_response.pdf [file pone.0238058.s001.pdf]

We thank the reviewers for their valuable comments on our article. Please find below our answers for each raised point. Note that all modifications in the article are shown in red.

Editor

- *"1. Please ensure that your manuscript meets PLOS ONE's style requirements, including those for file naming."*

  Answer: We have reviewed the manuscript and files, and made small corrections we understood were needed to fully meet the requirements, including rewriting section references to not use numbers, and changing the format of submitted images to TIFF.

- *"2. Were any images collected specifically for this study? Please provide additional details regarding participant consent to collect personal data and images. In the Methods section, please ensure that you have specified how consent was obtained and how the study met relevant personal data and privacy laws. If data were collected anonymously, please include this information. Please also indicate whether there was any ethical oversight over the study, and please include this information in both the Ethics statement and manuscript. Please also include information on how participants were recruited, and whether they consented to have their images shared publicly."*

  Answer: We have included an ethics statement to the Datasets section, describing how consent was obtained. As the dataset consists of face images, all volunteers who agreed to participate were required to sign a term of consent allowing the use of their images for research purposes. The term was approved by the Research Ethics Committee of the University of Campinas under the number 53035216.6.0000.5404. Following common practices, data is anonymized in the sense that no additional information that could lead to the identification of the subjects is stored with the images.

- *"3. We note that you have stated that you will provide repository information for your data at acceptance. Should your manuscript be accepted for publication, we will hold it until you provide the relevant accession numbers or DOIs necessary to access your data. If you wish to make changes to your Data Availability statement, please describe these changes in your cover letter and we will update your Data Availability statement to reflect the information you provide."*

  Answer: We have uploaded our dataset to the Zenodo repository, and reserved the DOI 10.5281/zenodo.3749309. The dataset is ready to be published upon acceptance of the manuscript.

- *"4. We note that Figure 2, 5, 6, 7 and 9 includes an image of a participant in the study."*

  Answer: We have obtained signed consent from all individuals depicted in the figure, and updated the manuscript accordingly to reflect that.

- *"5. We note that Figure 2, 5, 6, 7 and 9 in your submission contain copyrighted images."*

  Answer: We declare that all figures were created as part of this work, and we are the owners. All persons depicted in the figures have given written consent for publication.

Reviewer #1

- *"Strengths:*
  1) Authors have efficiently used SqueezeNet as the base of the training model which has around 1.2 million parameters that takes only 5MB of memory which is very suitable for a mobile device to operate."

  Answer: We thank the reviewer for the positive feedback and constructive comments to improve our work.

- *"2) The model structure is fully convolutional which made the input size and alignment much flexible and more accurate than AlexNet. All feature maps are flexible in size, but they kept the input shape as 3 x 227 x 227. And that gives one important flexibility. Scaling-down the pixels of the images does not lose much localisation information, rather it lets us free from taking long time to train the model."*

  Answer: The reviewer captured the rationale of our method. We thank the reviewer for the positive feedback.

- *"3) Authors used variable resolution face-patches of the images. They claimed that it reduces overfitting to user-specific characteristics. And thus the learning becomes more robust as the images do not remain tied to any single scale anymore."*

  Answer: The reviewer captured the rationale of our method. We thank the reviewer for the positive feedback.

- *"4) Through the experiment they found out that the camera specifications are very important here and while building the model this should be considered to avoid ending up with a biased model. And to overcome this problem, they came up with triplet-loss which also can be called intra-device triplet loss and spoof-loss which also can be called multi-objective loss instead of traditional cross-entropy loss."*

  Answer: The reviewer captured the rationale of our method. We thank the reviewer for the positive feedback.

- *"5) Authors expressed their experiment result using 5 common considered biometric presentation attack evaluation namely Attack Presentation Classification Error Rate (APCER), Bona fide Presentation Classification Error Rate (BPCER), Average Classification Error Rate (ACER), Half-total Error Rate (HTER) and Equal Error Rate (EER). This made their experiment much stronger."*

  Answer: Indeed we believe a proper validation is important to support all claims and we did our best effort to comply with top-level standards in the literature.

- *"Weaknesses:*
  1) In the datasets authors have used, the devices were used are a little old version. The latest phone they have used Moto G5 was released in 2017. But in those phones there was no face

detection technology. Rather face detection technologies are provided in the phones those were released very recently and obviously cameras on those phone are different. So those new phones like iphone 10, 11, galaxy s10 etc should have been given priority to acquire data. Also iPad should have been included in the device list."

Answer: We understand the concern raised by the Reviewer, and the importance of using the latest technology in our experiments. However, we can justify the choice of devices as follows: 1) Moto G5 was a very popular smartphone at the time data acquisition started. 2) Moto X Style XT1572, while relatively older, had a powerful frontal camera that was still representative of modern devices. 3) The images acquired by these devices had significantly different characteristics from one another to merit their use in our experiments, some of which were designed to test the generalization of PAD methods to different sensors. The face detection technology in more recent methods would work as an ally of our method so that it received as input a better detected face. Finally, we note that the methods we propose in the paper are NOT custom-tailored to particular devices and their application on more recent devices is definitely possible. Therefore we believe our contribution holds and many others will take advantage of the proposed methods while seeking for security for their devices.

- *"2) Authors didn't mention gender or racial diversity in the dataset. There should have been a proper diversity ratio in the dataset."*

  Answer: We think that the Reviewer's question is very relevant, as most similar datasets are collected in a low-variability environment. As a summary, we report that among the 45 users, 30 are male, 14 wear glasses, 13 have some kind of beard, and the ages range from 18 to 50 years old. We have added this information to the datasets section of the manuscript. Racial or ethnicity information was not collected, but it is clear that all continents are represented in the phenotypic makeup of the subjects. That said, we highlight the importance of the careful design of the five different illumination scenarios, which can have a higher impact in the study of the problem than subject variability.

- *"3) Authors should have shown not only images but how video streams perform with this model."*

  Answer: While we recognize that experimenting with videos would also have been interesting, we decided to focus on single static images for two reasons. First, we were motivated by the scenario where device unlocking should happen as quickly as possible, within the limitations of phone hardware, where waiting for and processing a stream of frames would necessarily increase the prediction latency and demand more resources from the device. Second, the use of dynamic sequences necessarily requires assumptions that were beyond the scope of this work. For example, how to define the kind and range of movements that users make when using a smartphone naturally? Instead of recording unrealistic sequences, we decided to focus on different combinations of users, faces, lighting, and background. Another point of interest here is that as our method works well for static images, its extension to dynamic images is not difficult as the minimum one could do is to consider the frames independently and train a classifier to perform the best (weighted) fusion possible.

- *"4) Authors should have shown what properties of other CNN structures are failing in mobile device environment while SqueezeNet architecture is winning the situation with proper use-cases*

and example situations."

Answer: In this work, we analyzed the impact of redefining the optimization problem, in terms of loss function and input. Note that the presented ideas could be useful in combination with ANY core-CNN architecture. We justify the decision of using SqueezeNet as the core architecture through the observation that it fulfills the following criteria: first, it is small and fast enough, given our hardware limitations; second it is already shown to work well for other difficult and general image classification problems. Comparing different core architectures, or presenting and justifying a completely new architecture would occupy valuable space in the manuscript without adding too much useful information as we propose methods that can be used with any core CNN.

- *"5) The main idea of this experiment was to differentiate between genuine access images and fake attack images. But ideally this experiment can be done not only on face images rather on images of other materials. They could show some results of experiments on the images other than face."*

  Answer: We understand the reviewer's suggestion. However, the problem of identifying face presentation attacks is per se a very difficult problem and different from the problem of identifying screen recaptures for other objects. What is the main difference? First of all, the main point of concern is the physical material. While with faces we are dealing with human skin with important properties we can explore during an attack (texture changes, smoothing properties, wrinkle marks before/after recapture) for the general problem of screen recapture, this is a whole new story as the objects would be way too different in terms of materials. Therefore, these two areas are considered separately in the literature. While presentation attack detection literature normally focuses on understanding the effects of light and texture on recaptured faces, normally the screen recapture literature tries to understand the lack of alignment of hard surface objects and aliasing properties. See some references below for general screen-recapture to check how different these problems are.
  [1] https://www.semanticscholar.org/paper/Modelling-radial-distortion-chains-for-video-Scarzanella-Dragotti/eb42ccbd915fb69f341a3667192c18fb6f986b83
  [2] https://ieeexplore.ieee.org/document/7010054

Reviewer #2

- *"This paper presents a CNN-based algorithm to detect face presentation attacks, specifically for mobile devices. The idea is to use patches of varying resolution. This not only increases the number of available examples but also solves the overfitting issues. The authors also introduce a multi-objective loss function to reduce inter-device confusion. A comprehensive evaluation has been conducted on RECOD-MPAD, a new dataset tailored to the mobile-device setup. The results show that the proposed method achieves better results compared with state-of-the-art solutions.*
  Strength:
  1: The paper is clearly written and well structured.
  2: A new dataset is developed. It will be great if the authors can make it publicly available.
  3: Evaluation is comprehensive and well justified."

  Answer: We thank the reviewer for the positive feedback and for the constructive comments toward improving our work.

- *"Weakness:*
  Novelty is somehow limited. My understanding is that the authors merely apply the existing SqueezeNet architecture to the mobile scenario. The authors claim that the dataset will be made available along with the article publication. It cannot be verified at the review stage."

  Answer: The main contributions of this work are twofold: first and foremost, the definition of a loss function that takes into account inter-device variabilities and second, the proposal of training procedure using a single network with variable crop sizes, without the necessity of rescaling inputs. In principle, these ideas are independent of the convolutional core, and could be applied to arbitrary architectures being an important advance in the presentation-attack detection literature. Note that properly designing a loss function for this difficult problem is not trivial. It needs to account for several nuances of the problem and the one we propose in the paper is the result of a two-year exploration of different problem formulations. In the end, it might seem trivial to see a formula and downplay it but it is a very important add-on to any network designed for the problem and that is why this paper has value: the proposed loss function can be applied in different network formulations. In addition to the function itself, the finding of a possible training procedure that relies on image patches of variable size is an advance in the literature that had established before that ALL inputs should be the same size. Perhaps that was true for existing loss functions at the time but with problem-tailored functions like the one we propose it is better to consider variable patch size as we consider. These two contributions mean an advance in the field as the extensive battery of experiments show.
  In addition, we opted to use SqueezeNet as the core network in our experiments because it is already established, saving the space and experiments that would be necessary to justify other architectural decisions in the manuscript. We present the results of the traditional approach of using a single rescaled face crop as input, and training with cross-entropy loss, as one of our baselines.
  Finally, additional contributions of our work (also listed in the introduction) are: the unique dataset that was collected for the experiments and will be freely available upon acceptance; an extensive study of error cases, which tests generalizability to different lighting, attack types, and sensors; and a method that explores a key characteristic of the target use case to further increase

the discriminability of the model. We thank the Reviewer for recognizing the value of our dataset, which we confirm will be made publicly available upon publication.

- *"Minor:*
  Page 2 line 42 - 49: missing section numbers.
  Page 6 line 220 - 223: missing section numbers."

  Answer: We have reviewed and updated all section references in the manuscript.
