# Supplementary material for: Detecting face presentation attacks in mobile devices with a patch-based CNN and a sensor-aware loss function
Source: PLoS One. 2020 Sep 4;15(9):e0238058. doi: 10.1371/journal.pone.0238058 (PMC7473524; doi:10.1371/journal.pone.0238058)
Supplement: Attachment — Submitted filename: 2020_biolive_pad_plosone_response.pdf [file pone.0238058.s002.pdf]

We thank the reviewers for their comments on our article. Please find below our answers for each raised point. Note that all modifications in the article are shown in red.

Reviewer #1

- *'The authors have addressed all comments satisfactorily. I recommend acceptance. This could be an important paper in the community of computer vision and security."*

  Answer: We thank the reviewer for the positive feedback.

Reviewer #2

- *"1. Have the authors made all data underlying the findings in their manuscript fully available? Reviewer #2: No"*

  Answer: The proposed dataset is now available in a public repository, only restricted by a simple End-User License Agreement (EULA), as it is required by the Research Ethics Committee of the University of Campinas that approved the collection of the data under the number 53035216.6.0000.5404.
  The dataset and the EULA can be accessed in the following public link: `https://zenodo.org/record/3749309#.Xw0xIJMzY8O`.

- *"2. Thanks the authors for the contribution clarification. Another concern I have after reading the revision is that the technique proposed in the paper is limited to images captured by traditional cameras. However, with the development of new technologies, such as face ID, which creates a depth map of faces and also captures an infrared image, the proposed approach will soon be obsolete. There is no doubt that new hardware can achieve better accuracy and privacy. The authors can argue that the proposed approach can be used in low-end smartphone. Other than that, could the authors provide any other insights?."*

  Answer:
  The objective of the presented research is to enrich the understanding of how a purely software-based method can be used for presentation attack detection, therefore we present a solely data-driven approach trained with multi-resolution patches and a multi-objective loss function crafted specifically to the problem. This is the main reason why the method is limited to images captured by traditional cameras, as the reviewer pointed out.
  Apart from being applicable to low-end devices, which do not contain advanced sensors, our method could be combined or extended with different types of inputs, including depth information as well, thus taking advantage of new technologies. Furthermore, the proposed loss function is generic enough to be applied to other PAD problems, forcing the genuine-access examples from the same capturing device to be more compactly located in the learned feature space, while reducing inter-device confusion.
  We have added this discussion in the Conclusions.
